# Hypertension is associated with better brain health outcomes in older-age asymptomatic carotid disease populations - A Multi-Cohort Study

**DOI:** 10.21203/rs.3.rs-10731777/v1

**Published:** 2026-09-10

**Authors:** Craig Weinkauf, Summan Zahra, Alexander Hong, Juan Arias, Scott French, Haley Wiskoski, Ivan Luu, Erin Oxnam, Elizabeth Orobiyi, Madeline Ally, Cris Escareno, Gavin Culwell, Emma Heitkamp, Franchell Vazquez, Paola Vidana, Xana Waughman, Madison Hillis, Kevin Johnson, Dedrick Jordan, Georg Hishaw, Edward Bedrick, Francesca Vitali, Raza Mushtaq, Maria Altbach, Jeremy Pruzin, Timothy Hughes, Gene Alexander, Tze-Woei Tan

**Affiliations:** The University of Arizona; University of Arizona; University of Arizona

## Abstract

**Background:**

Blood pressure (BP) management is highly relevant for cardiovascular and brain health outcomes. Although clinical guidelines are trending towards increasingly strict BP control, some discordant results evaluating dementia-related outcomes indicate that more research is needed. Asymptomatic extracranial carotid atherosclerotic disease (aECAD), a hallmark cerebrovascular disease affecting > 10% of those over the age of 60, may represent a key subpopulation unaccounted for in previous studies.

**Methods:**

Using our Carotids and Minds prospective study and TriNetX, a US nationwide medical record database, we evaluated the association of hypertension status with dementia-related outcomes in adults ≥ 50 years of age with and without aECAD. We investigated secondary effects of age, systolic BP (SBP) ranges, hypertension medications, cardiovascular outcomes, and treatment of aECAD with carotid endarterectomy (CEA).

**Results:**

After controlling for relevant confounders, hypertension was significantly associated with higher cognitive scores (β = 0.47, p< .01), lower pTau217 (β= −0.52, p<.01) and lower AD (HR = 0.56 [0.52–0.61]), non-AD dementia (HR = 0.66 [0.63–0.68]), and MCI risk (HR = 0.69 [0.64–0.74]) in patients with aECAD. Effect sizes were moderate to large and congruous across both cohorts, with strongest risk reduction in aECAD patients 81 + years of age. CEA mitigated these protective associations, and significantly higher risk of ADRD was seen in hypertensives without aECAD. Still more, a SBP range of 140–159 mmHg optimized protective effects with no significant increase in major cardiovascular events and death.

**Conclusion:**

In summary, hypertension is associated with lower risk of dementia-related outcomes in aECAD patients. This promotes the need for future clinical trials focusing on individualized BP goals and addressing cerebrovascular disease. More broadly, these data further highlight cerebrovascular disease as relevant in brain health and aging.

## INTRODUCTION

Approximately 6.9 million people in the US above 65 years of age are living with Alzheimer’s disease (AD).^1^ Vascular risk factors (hypertension [HTN], diabetes, smoking, hyperlipidemia) ^2-4^ and vascular diseases (including asymptomatic carotid atherosclerotic disease [aECAD]), ^5-7^ contribute to Alzheimer’s disease and related dementias (ADRD) risk. HTN is particularly well established as a risk factor for ADRD^8^ with the strongest data linking mid-life HTN to late-life cognitive impairment and dementia.^9,10^ Because of this, numerous clinical studies have explored blood pressure (BP) reduction as a strategy to mitigate ADRD risk.

Evaluation of BP reduction in both cross-sectional and longitudinal cohort studies have had inconsistent results regarding dementia-related outcomes. ^11-15^ Randomized clinical trials (RCT) examining the role of BP control in cognitive function and dementia risk have likewise yielded some conflicting results ^15,16^. The ACCORD-MIND trial showed that among individuals with high risk for cardiovascular disease, intensive BP control was not effective in reducing cognitive decline.^16^ In contrast, in the SPRINT-MIND trial, intensive BP control decreased the risk of mild cognitive impairment (MCI) and decreased accumulation of white matter disease;^15^ however, intensive BP control had no significant effect on incident dementia (possibly a limitation of early trial termination and inadequate follow-up) ^14^ and also resulted in *decreased* total gray matter and hippocampal volumes.^17^ We summarized key findings from relevant RCTs in Supplemental Table 1^14,16,18-21^. Overall, there is a clinical and research trend towards stricter BP management to improve cardiovascular and brain health outcomes. However, there may be individuals that do not benefit from intensive BP control.

HTN is also a primary modifiable risk factor for aECAD.^22,23^ Clinical guidance for BP control in aECAD population is largely extrapolated from broader cardiovascular risk management trials, and the 2017 American Heart Association/American College of Cardiology guidelines lowered the BP threshold to < 130/80 mmHg, matching that of the general population.^24^ It is notable that these guidelines do not address whether cerebral perfusion and/or cognitive outcomes are relevant to consider in this population.

aECAD and aging have both been associated with decreased cerebral perfusion,^25-27^ which has been associated with cognitive decline and increased dementia risk.^28^ In addition, several longitudinal studies have shown that lower BP in the elderly (> 70 years of age) and frail is associated with poorer dementia- related outcomes ^11,29-31^. In contrast to age, aECAD status has not been accounted for in studies looking at BP management and dementia-related outcomes. We think that aECAD may be an important factor because aECAD is associated with an increased risk for AD and related AD pathology,^6,32,33^ is related to an increased risk for cerebral hypoperfusion,^25^ and occurs in 10% or more of the population over 60 years of age.^34^

For these reasons, we hypothesized that HTN may interact with aECAD, resulting mitigating effects for dementia-related outcomes. To study the interaction of HTN and aECAD on dementia-related outcomes, we evaluated two complementary cohorts. This included 1) analysis of our own prospective Carotids and Minds (CAM) observational study, which was designed to study the associations of aECAD with dementia-related outcomes^35^, and 2) the USA-wide electronic medical record database TriNetX with a longitudinal cohort study design. Dementia-related outcomes were selected a priori based on relevant data available in each database and were reported fully. With the large power of the TriNetX database, we explored several secondary hypotheses regarding the relevance of age, bilateral versus unilateral aECAD, the effects of carotid endarterectomy (CEA), the benefits of specific systolic BP (SBP) ranges, and the potential cardiovascular risks associated with elevated SBP.

## METHODS

### Primary Cohort, CAM

The first 182 participants enrolled in the prospective Carotids and Minds (CAM)^36^ clinical study were used for this analysis. CAM is a longitudinal observational study that evaluates the effect of vascular risk comorbidities on cognitive impairment and AD pathology. Adults aged 50–85 years were recruited from surgery or cardiology academic and community health clinics in Tucson, Arizona from 2022–2024. Individuals were eligible if they had a clinical diagnosis of asymptomatic extracranial carotid atherosclerotic disease (aECAD) and/or at least two vascular risk factors, including smoking, HTN, hyperlipidemia, or diabetes mellitus. aECAD was defined as having carotid stenosis ≥ 50% without a history of stroke or transient ischemic attack (TIA) in the last six months. Exclusion criteria included a clinical diagnosis of dementia, major depression, neurological disorders, end-stage renal disease, heart failure, or terminal cancer. Demographic information and past medical history were determined based on participants’ self-report (reviewed by study coordinators with participants) and medical records review. All study procedures were approved by the Institutional Review Board at the University of Arizona. All participants gave written informed consent.

**Carotid Stenosis Evaluation**: Percent carotid stenosis was adjudicated by a panel of three experts (CW, RM, KJ) evaluating high-resolution, multi contrast MRI data as described previously ^37^. Highest percent stenosis of the two carotid arteries was used for analyses. **White Matter Lesion (WML) Volume Quantification**: The WML volumes (mm^3^) were segmented using our in-house developed software, a 3D orthogonal deep learning convolutional neural network. ^38^ WML volume data were missing in some participants (n = 18) who could not complete the scan session. **Neurocognitive Testing**: The Montreal Cognitive Assessment (MoCA), a rapid and sensitive screening cognitive instrument, was used to assess cognition within CAM cohort under the guidance of a trained neuropsychologist (GA). **pTau217 and Apolipoprotein E (APOE) Genotyping**: Plasma pTau217 and *APOE* genotypes were quantified through collaboration with the Biomarker Assay Laboratory at the National Centralized Repository for Alzheimer’s Disease (NCRAD) as described previously

### Secondary Cohort, TriNetX

#### Data Source

This cohort study analyzed data from TriNetX Research Network, a federated, real-time platform, which collects deidentified patient-level data from electronic health records from over 120 million individuals across 71 U.S. health care organizations, including academic and non-academic medical centers as well as affiliated satellite sites. TriNetX adheres to the HIPAA guidelines and does not pool raw patient-level data into a central repository. Instead, it enables real-time querying of deidentified patient information across its network while ensuring that data remain within the originating institutions. Patient-level data were analyzed using built-in statistical tool on the TriNetX platform. Further details about the database can be found online.^39^

#### Study Population

A retrospective cohort of adult patients aged ≥ 50 years with at least two years of observation prior to index event within the platform were used. Patients with a history of stroke, TIA, carotid artery stenting, carotid endarterectomy, MCI, AD and non-AD dementia on or before index event were excluded. Patients with missing data were also excluded. The main study population was divided into two cohorts, i) aECAD and ii) No ECAD. All diagnoses were identified using *International Statistical Classification of Diseases, Tenth Revision* (ICD-10) details of which are provided in Supplemental Tables 2–3.

1. Within the aECAD cohort, the index event was defined as the first diagnosis of aECAD after age of 50 years (or age ≥ 60 for the 60–70-year sub-group and similarly for other age groups.)
2. Within No ECAD cohort, all individuals had at least one outpatient clinic visit and no diagnosis of ECAD. Index event was defined as the first outpatient clinic visit after age of 50 years on the same day as the entry into the cohort for an aECAD patient. This method ensured temporal alignment between No ECAD and aECAD cohorts. All individuals were defined to have “No ECAD” on or prior to the index event.

Both cohorts were further stratified based on the history of hypertension on or prior to index event into hypertensive (HTN) and non-hypertensive (No HTN) groups. Within aECAD and No ECAD cohorts, HTN and No HTN groups were 1:1 propensity score matched for age, sex, race, ethnicity, ischemic heart diseases, diabetes mellitus, hyperlipidemia, atrial fibrillation and flutter, nicotine dependence, alcohol related disorders, overweight and obesity, hearing loss, socioeconomic and psychosocial health hazards, body mass index (BMI), glycosylated hemoglobin, serum HDL cholesterol, serum LDL cholesterol, and serum triglycerides. Covariate selection was guided by clinical relevance including both major comorbidities and risk factors that could contribute to cognitive and cardiovascular outcomes in accordance with current research.

#### Sub-group Analyses

To assess the robustness of our findings and explore the relative risks and benefits associated with various exposure strata, we conducted additional analyses designed to address both biological plausibility and clinical heterogeneity in our aECAD cohort.

##### i) Age Stratification

aECAD patients were further stratified based on their baseline age into four groups: 50–60 years, 61–70 years, 71–80 years, and 81 + years. aECAD/HTN patients were compared to aECAD/No HTN patients within each age group. This approach was intended to account for the age-dependent trajectory of both vascular pathology and cognitive decline.

##### ii) Systolic Blood Pressure Stratification

Second, aECAD patients 81 + years were stratified into systolic blood pressure (SBP) categories using clinically meaningful cutoffs: 140–159 mmHg, 160–179 mmHg, and ≥ 180 mmHg compared to a reference group of aECAD/No HTN. Patients were required to have at least five distinct SBP values recorded prior to cohort entry to optimize stable SBP classification. These categories reflect guideline-relevant thresholds and were analyzed to evaluate a potential dose-response relationship between SBP and dementia outcomes.

##### iii) aECAD/HTN and Carotid Endarterectomy (CEA)

We performed a subgroup analysis of aECAD patients who had undergone CEA to determine whether surgical revascularization modified the association between blood pressure and dementia.

##### iv) Bilateral vs Unilateral aECAD

To explore the impact of anatomical burden of disease, we compared outcomes between patients with unilateral versus bilateral aECAD. Because bilateral disease may result in more profound cerebral hypoperfusion, this analysis was performed to evaluate whether laterality of stenosis influenced the relationship between HTN and cognitive risk.

##### v) Use of Anti-hypertensive medications

We conducted a medication-based subgroup analysis to evaluate the potential modifying effects of blood-brain barrier (BBB)-crossing antihypertensive agents on cognitive outcomes. These agents have been proposed to exert direct neuroprotective effects through central mechanisms beyond systemic blood pressure control.^40^ To interrogate this hypothesis, we performed several comparisons. First, we compared patients prescribed BBB-crossing antihypertensives to those receiving non-BBB-crossing antihypertensive agents, matching for other non-BBB crossing treatment. This allowed us to isolate the effect of BBB penetration from general antihypertensive use. Second, we compared patients on BBB-crossing agents to those not receiving any antihypertensive therapy, to assess whether BBB-specific agents conferred protection above untreated HTN. For all medication subgroups, exposure was defined as medication prescription recorded within the 6 months prior to the diagnosis of aECAD, to ensure temporal proximity and pharmacologic relevance.

### Exposures and Outcomes

The primary exposure was defined as history of HTN within aECAD and No ECAD groups. Primary outcomes included MCI, AD and non-AD dementia. Secondary outcomes included major adverse cardiovascular events (MACE), myocardial infarction (MI), heart failure, chronic kidney disease (CKD), stroke and all-cause mortality.

### Statistical Analyses

T-test or χ^2^ square tests were used to test the significance of the differences on continuous and categorical variables. Non-parametric tests were used when normality criteria were not met. All statistical tests were two-sided, with P-values less than 0.05 considered statistically significant.

#### a) CAM

Multivariable linear regression was used to assess effects of HTN, age, sex, education, race, ethnicity, and other vascular risk comorbidities on cognitive outcome (MoCA) and AD pathology (pTau217). pTau217 levels were log-transformed prior to linear regression analyses due to skewness; post log-transformation was normally distributed. WML volumes also were transformed to a logarithmic scale by using the equation log (WML volume + 0.001) taking into consideration skewed distribution and participants having no WML. β estimates for all regression models were standardized and models were tested for multicollinearity using variance inflation factor (VIF), a VIF < 1.2, indicating no multicollinearity. All statistical tests were two-sided, with P-values less than 0.05 considered statistically significant. All analyses were conducted in R version 4.3.1.

#### b) TriNetX

Propensity score matching was performed using 1:1 nearest-neighbor matching with a caliper of 0.1 pooled SD of the logit of the propensity score. An SMD < 0.1 for matched covariates was considered indicative of adequate balance. Cox proportional hazards models were used to estimate hazard ratios and 95% CIs for all outcomes. The proportional hazards assumption was tested using scaled Schoenfeld residuals in R studio (version 3.2-3). Time-to-event outcomes were analyzed using Kaplan-Meier survival curves and compared using the log-rank test. Statistical analyses were conducted using the analytic tool on the TriNetX platform.

## RESULTS

### Primary Cohort – CAM

#### Demographics

The CAM study included 167 participants excluding screening failures and missing data (n = 15). (Supplemental Fig. 1) Mean age was 72 ± 7 years, 57% were males, mean education was 14.5 ± 2.9 years, and most participants had at least some college experience. CAM participants were predominantly White (96%) with 11% of Hispanic/Latino ethnicity. Seventeen percent of the participants had a remote history of stroke, 37% had coronary artery disease, and 9% had a history of TIA. Prevalence of No ECAD and aECAD was 36% and 64%, respectively. (Supplemental Table 4).

#### HTN and Cognition

Within the whole CAM cohort, HTN was associated with increased MoCA scores that trended toward statistical significance (P=.07). Furthermore, HTN was associated with significantly higher MoCA scores (P=.01) in those with aECAD, but not without aECAD (P=.64). (Fig. 1) Using a linear regression model adjusting for age, sex, education, diabetes, stroke and *APOE ε4* (significant predictors for cognition in all models), there was a significant interaction between HTN and aECAD (HTN x aECAD, **β**=0.71, CI: 0.13–1.28, P=.017). (Fig. 1). Within sub-group analyses, the association between HTN and MoCA scores remained significant within aECAD group after adjusting for age, sex, education and *APOE ε4 (***β**=0.49, P<.01), vascular comorbidities (**β**=0.48, P<.01) and white matter lesion volumes (**β**=0.48, P=.02).(Fig. 2) However, multivariable linear regression models within the No ECAD group showed that HTN was not significantly associated with MoCA scores. (Supplemental Fig. 2a-c).

#### HTN and pTau217

Within the whole CAM cohort, HTN was associated with decreased plasma pTau217 levels (P=.02). In the aECAD group, HTN was associated with significantly lower pTau217 levels (P<.01) but not in the No ECAD group (P=.69) (Fig. 3). Using a linear regression model adjusting for age, sex, education, diabetes, stroke and *APOE ε4* (significant predictors for cognition in all models), the interaction between HTN and aECAD for pTau217 as an outcome was not significant. (HTN x aECAD, **β**= −0.48, CI: −1.10–0.14, P=.12) (data not shown). However, in sub-group analyses, models within the aECAD group showed that HTN was associated with significantly decreased pTau217 levels independent of age, sex, education and *APOE ε4 (***β**= −0.43, P=.02), vascular comorbidities (**β**= −0.51, P=.009) and white matter lesion volumes (**β**= −0.45, P=.046) (Fig. 3). Multivariable linear regression models within the No ECAD group showed that HTN was not significantly associated with pTau217 levels. (Supplemental Fig. 2d-f).

#### Anti-Hypertensive Medications

Additional analyses were conducted to evaluate whether the association between HTN and cognitive performance was confounded by anti-hypertensive medication use. After adjustment for treatment status, HTN remained significantly associated with higher MoCA scores (**β**= 0.40, P=.03), independent of age, sex, education, *APOE* e4 and vascular comorbidities. Anti-hypertensive medication use alone was not significantly associated with MoCA scores (**β**= 0.23, P=.24).(Supplemental Fig. 3a) Furthermore, anti-hypertensive medications were classified into blood brain barrier (BBB) crossing medications (fosinopril, lisinopril, ramipril, telmisartan) and non-blood brain barrier (non-BBB) crossing medication (all other antihypertensive medications).^41^ Use of BBB-crossing medications was not significantly associated with MoCA scores (**β**= 0.40, P=.116) while HTN remained independently associated with higher MoCA scores (**β**= 0.38, P=.04). (Supplemental Fig. 3b) Similarly, the association between HTN and lower levels of pTau217 persisted after adjustment for anti-hypertensive medications use and BBB-crossing medications specifically. (Supplemental Fig. 3c-d)

### Secondary Cohort – TriNetX

#### Demographics

A total of 811,686 aECAD patients were included (Supplemental Fig. 4). After propensity score matching, 215,336 patients remained in HTN/No HTN group (age 68.3 ± 8.7 years; 49% male). The mean follow-up duration was similar between groups (4.2 ± 3.7 years for aECAD/HTN vs 4.2 ± 3.9 years for aECAD/No HTN) (Supplemental Table 5). A total of 2,608,333 No ECAD patients were included (Supplemental Fig. 4). After propensity score matching, 711,674 patients remained in each cohort (age 66 ± 8.6 years; 43% male). The No ECAD/HTN cohort was followed for 3.9 ± 3.2 years compared to 3.8 ± 3.2 years for the No ECAD/No HTN cohort (Supplemental Table 6).

#### AD, Non-AD dementia and MCI risk

Among aECAD patients, those with HTN had significantly lower risks of AD (HR 0.93[0.88–0.98]), non-AD dementia (HR 0.97[0.95–0.99]), and MCI (HR 0.95[0.91–0.99]) compared to those without HTN. In contrast, among No ECAD patients, HTN was associated with significantly higher risks of AD, non-AD dementia and MCI compared to those without HTN. (Fig. 4)

#### Age and SBP stratification in aECAD

HTN status did not show any significant differences in risk for MCI, AD, and non-AD dementia in the 50–60 age group. However, MCI, AD, and non-AD dementia risk decreased significantly in 61–70, 71–80 and 81 + age groups with HTN compared to without HTN. The 81 + group with aECAD exhibited the greatest risk reduction for AD (HR 0.56 [0.52–0.61]), non-AD dementia (HR 0.66 [0.63–0.68]), and MCI (HR 0.69 [0.64–0.74]) (Fig. 5a) and improved survival for all three MCI/dementia outcomes. (Log-rank p<.0001, Supplemental Fig. 5). In contrast, among patients aged 81 + without aECAD, those with HTN had significantly higher risks of AD (HR 1.93 [1.77–2.11]), non-AD dementia (HR 1.68 [1.60–1.76]), and MCI (HR 1.45 [1.32–1.59]) compared to those with No HTN. (Data not shown)

We evaluated three hypertensive SBP ranges in patients with aECAD (SBP 140–159, 160–179, and ≥ 180 mmHg) compared to aECAD patients without HTN. The SBP ranges each had significantly lower HR for AD, non-AD dementia and MCI except for SBP ≥ 180 mmHg, which was not significant for MCI. SBP 140–159 mmHg had overall the lowest HR of the three blood pressure ranges. (Fig. 5b)

#### Carotid Endarterectomy and aECAD laterality

We hypothesized that if the protective effect of HTN is related to atherosclerotic blockage of aECAD, then in those with aECAD and CEA, the protective effect of HTN would be mitigated. We evaluated this in the age groups where HTN effects were strongest. In both the 71–80 and the 81 + years of age cohorts, HTN had no significant effect on AD, non-AD dementia, or MCI in those with CEA compared to those with aECAD without HTN. (Table 1)

Because bilateral more than unilateral aECAD could further compromise cerebral perfusion, we hypothesized that aECAD patients with unilateral disease would have lower HR for MCI/dementia outcomes compared to those with bilateral disease. Among aECAD patients with HTN, those with unilateral compared to bilateral aECAD had lower risks of AD (HR 0.82 [0.75–0.90]), non-AD dementia (HR 0.86 [0.83–0.90]), and MCI (HR 0.91 [0.85–0.98]). (Supplemental Table 7)

#### Use of Anti-Hypertensive Medications

Similar to CAM analyses, we explored whether HTN effects were secondary to anti-hypertensive medicine usage. We focused on BBB-crossing medications as they have been shown to associate with dementia outcomes in previous studies.^41^ BBB-crossing medications did not have significant effects on relevant outcomes (Supplemental Table 8).

#### HTN and cardiovascular Risk

Partitioning the 81 + group with aECAD by SBP ranges, SBP 140–159 mm Hg was not associated with significant differences in the risk of MACE, stroke, CKD, HF, MI or all-cause mortality compared to the reference group (81+, aECAD with SBP 130–139 mmHg). In contrast, SBP of 160–179 mmHg and SBP ≥ 180 mmHg were associated with significantly higher risks of all evaluated cardiovascular risks and mortality, except for HF in the SBP of 160–179 mmHg group, which was statistically equivalent (Supplemental Table 9).

## DISCUSSION

In this multi-cohort study, we found that in patients with aECAD, HTN is associated with significantly higher cognitive scores, decreased plasma pTau217 levels and decreased risk of MCI, AD, and non-AD dementia. These effects of HTN were strongest in the aECAD cohort 81 + years of age, were stronger when patients had unilateral vs bilateral aECAD, were independent of anti-hypertensive medications, and were mitigated if patients had treatment with CEA. Furthermore, we found that for aECAD patients 81 + years of age, SBP ranges of 140–160 mmHg were associated with lower ADRD risk yet did not significantly increase cardiovascular risks (including MACE, MI, heart failure, CKD, stroke, and all-cause mortality). Importantly, there has been established and emerging support for implementing increasingly strict BP goals, yet these data demonstrate that a more nuanced, patient-specific approach may be needed to optimize brain health benefits across older adult populations with prevalent cerebrovascular disease.

As summarized in Supplemental Table 1^16,18-21,42^, previous clinical trials have shown mixed results related to whether strict SBP control is beneficial for dementia-related outcomes. Importantly, no studies we could find, evaluated carotid stenosis or related cerebrovascular markers of carotid disease or measures of cerebral perfusion. These data argue that this is an important omission because HTN has opposite effects in patients with aECAD compared to those without. Further, aECAD is a common disease (occurring in 10% of the population > 60 years of age)^34^ and is associated with increased AD and non-AD dementia risk.^6^ It may be that some conflicting results in previous studies result from this unaccounted variation in aECAD and aging prevalence.

The prospective CAM study was designed to evaluate the association of aECAD with ADRD pathology independent of other vascular risk factors. Findings of higher MoCA scores and lower pTau217 levels associated with HTN-aECAD led to our hypothesis that elevated BP levels may reflect a protective physiology of counteracting cerebral hypoperfusion that can be seen in this population.^25^ This model and other potential mechanisms can hopefully be more thoroughly tested in our future work and other studies. Although the CAM cohort has strengths because of the extensive prospective validation/evaluation of carotid stenosis severity and ADRD outcomes, it is limited by being a relatively small (n = 167) single-center study, not specifically designed to study BP and associated BP parameters. Because of this, we sought to understand whether these findings extend beyond the CAM cohort and obtain added power to ask questions about specific SBP ranges and aECAD interactions.

Evaluating a large, USA-wide cohort (TriNetX), we found moderate to large effects between HTN and aECAD associated with MCI/ADRD risk. Among patients with aECAD, HTN was consistently associated with lower risk of AD, non-AD dementia, and MCI. In contrast, in those without aECAD, HTN was consistently associated with higher risk of AD, non-AD dementia, and MCI. The effect size of the HTN’s association increased with increasing age (strongest in the 81 + sub-group).^28,25^. Higher BP needed to counteract hypoperfusion in aging frail populations was also postulated by a recent study where HTN was associated with lower dementia risk. Our findings can be explained by worsening hypoperfusion seen with aging^28^ and aECAD^25^ (thus resulting in two perfusion insults to the brain), as well as by the increased overall risk of MCI/dementia-related outcomes with increasing age.

Although BBB-crossing medications have been associated with improved cognitive outcomes^41,43^, we did not find this was a relevant confounder in our analyses (similar results were seen in CAM). To understand the influence of SBP ranges, since increased SBP results in increased ADRD risk (for example, through increased cerebral small vessel disease) and increased cardiovascular or stroke risk^22^, we focused our analyses on the 81 + cohort where the strongest effects were seen. An SBP 140–159 mmHg conferred similar (or even better) benefits of ADRD risk reduction compared to those with higher recorded SBP. Similar investigations of SBP were performed with the CAM cohort (data not shown), but no significant results were found differentiating SBP groups, which may be related to limited power. Using the same SBP groupings, we also found that cardiovascular risks (MACE, MI, heart failure, CKD, stroke and all-cause mortality) increased in groups with HTN. However, the SBP 140–159 mmHg group had non-significant change of all cardiovascular and mortality risks. The RETREAT-FRAIL trial showed similar findings.^44^ These results suggest a potential “permissive HTN” range that balances risks and benefits for SBP.

Our findings that CEA can mitigate the protective effects of HTN complement previous data showing that CEA can mitigate ADRD risk, ^45^ but have complex clinical implications. One interpretation is that CEA would enable strict SBP goals; as a counter-point, this finding also raises the potential that simply allowing elevated SBP could protect patients with aECAD, obviating a need for CEA to prevent MCI/dementia outcomes in this population. Future studies could further explore these relationships.

Limitations of the TriNetX include the retrospective cohort design, which precludes causal inferences. Reliance on ICD-10 codes introduces the possibility of misclassification, undercoding, or missing data, leading to residual confounding. Outpatient SBP measurements may not capture variability or long-term trends, but an attempt to address this was done by requiring five SBPs documented to enter into the given SBP group. Attempts to mitigate limitations of TriNetX was made by using a longitudinal cohort study design with extensive propensity score matching to minimize differences and unmeasured confounding.

This work raises important clinical considerations by demonstrating that HTN may benefit patients with aECAD for dementia-related outcomes. Confidence in these findings can be derived from consistent aECAD/HTN trends across two cohorts, no other literature contradicting these results, a plausible physiological mechanism (cerebral perfusion), methodology in agreement with other analyzed outcomes (cardiovascular risk outcomes reported herein are in agreement with broader literature), and multiple sensitivity analyses (age groups, anti-hypertensive medications, bilateral/unilateral carotid disease, SBP ranges, and CEA). However, clinical care for this population is complicated because there are risks associated with HTN that include devastating outcomes like stroke and MACE. We think these data promote future clinical trials to test a more personalized approach to BP management accounting for cerebrovascular disease burden and increasing age.

## CONCLUSION

In summary, current ongoing management of BP is trending toward stricter goals with many clinical trials evaluating BP goals of < 120 mmHg. Our analyses demonstrate that this strategy may be associated with harm in patients with aECAD, especially in those with advancing age. Understanding the interaction between aECAD and HTN seems highly relevant because it will help guide management of a large subpopulation of patients and because inclusion of patients with aECAD in clinical trials could result in underestimation of BP control in those without aECAD (as these data also show). Finally, we want to highlight that this work is of further importance because it raises awareness of the potential role of cerebral perfusion physiology in ADRD risk and BP management.

## Supplementary Material

This is a list of supplementary files associated with this preprint. Click to download.

• aECADHTNSupplementalCommMed.docx

## Figures and Tables

**Figure 1:**
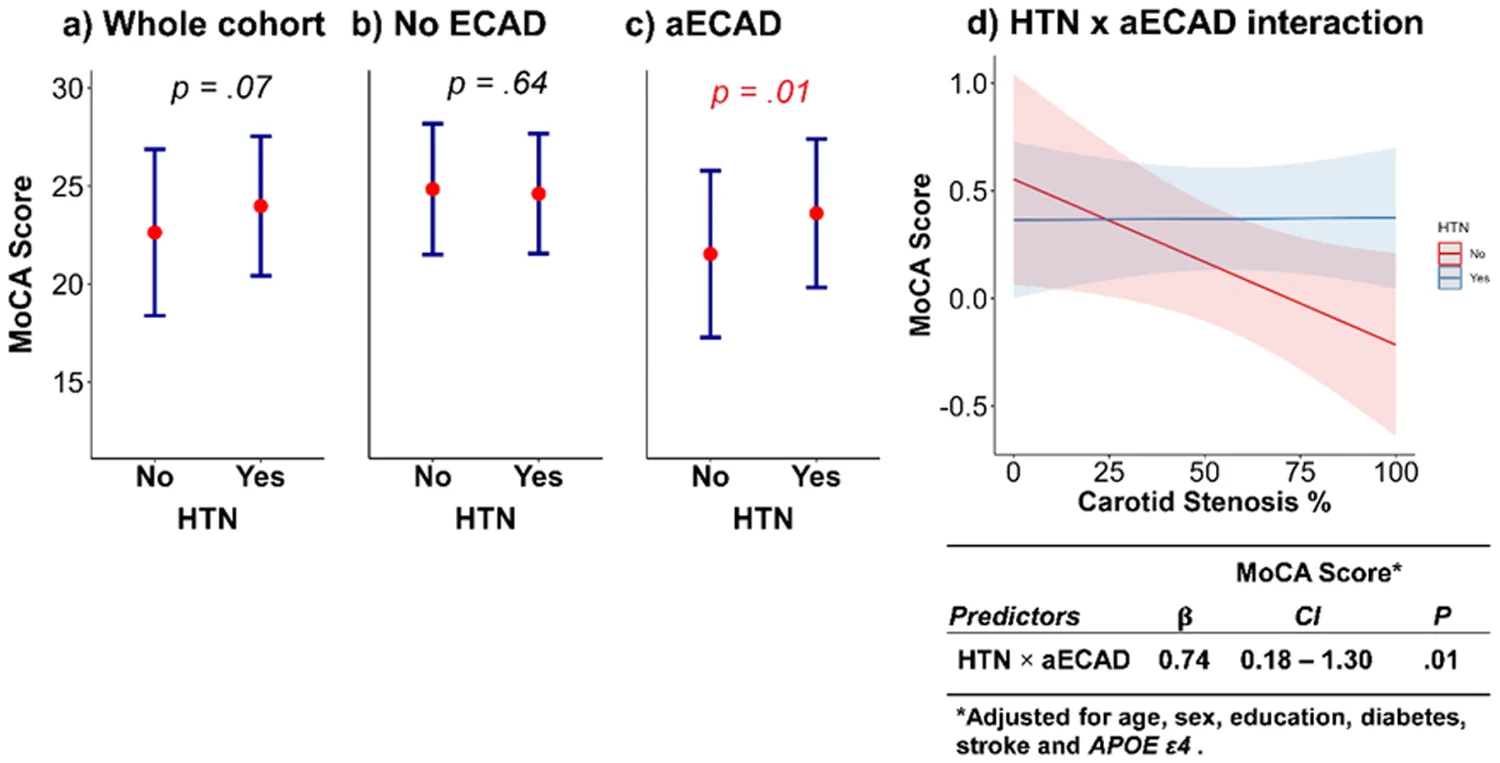
Parts a-c. The effect of HTN on cognition is moderated by aECAD in the CAM cohort. **a-c)** Mean MoCA scores were compared between those with HTN and without based on aECAD status. Mean differences were evaluated using Wilcoxon test. **d)**An interaction model was run between HTN and aECAD with an outcome of MoCA score; the model was adjusted for age, sex, education, *APOE ε4 allele,* diabetes, hyperlipidemia, and stroke*.* Abbreviations: HTN, history of HTN; aECAD, asymptomatic extracranial carotid atherosclerotic disease; **β**, standardized beta estimate; CI, confidence interval; *P*, p-value.

**Figure 2:**
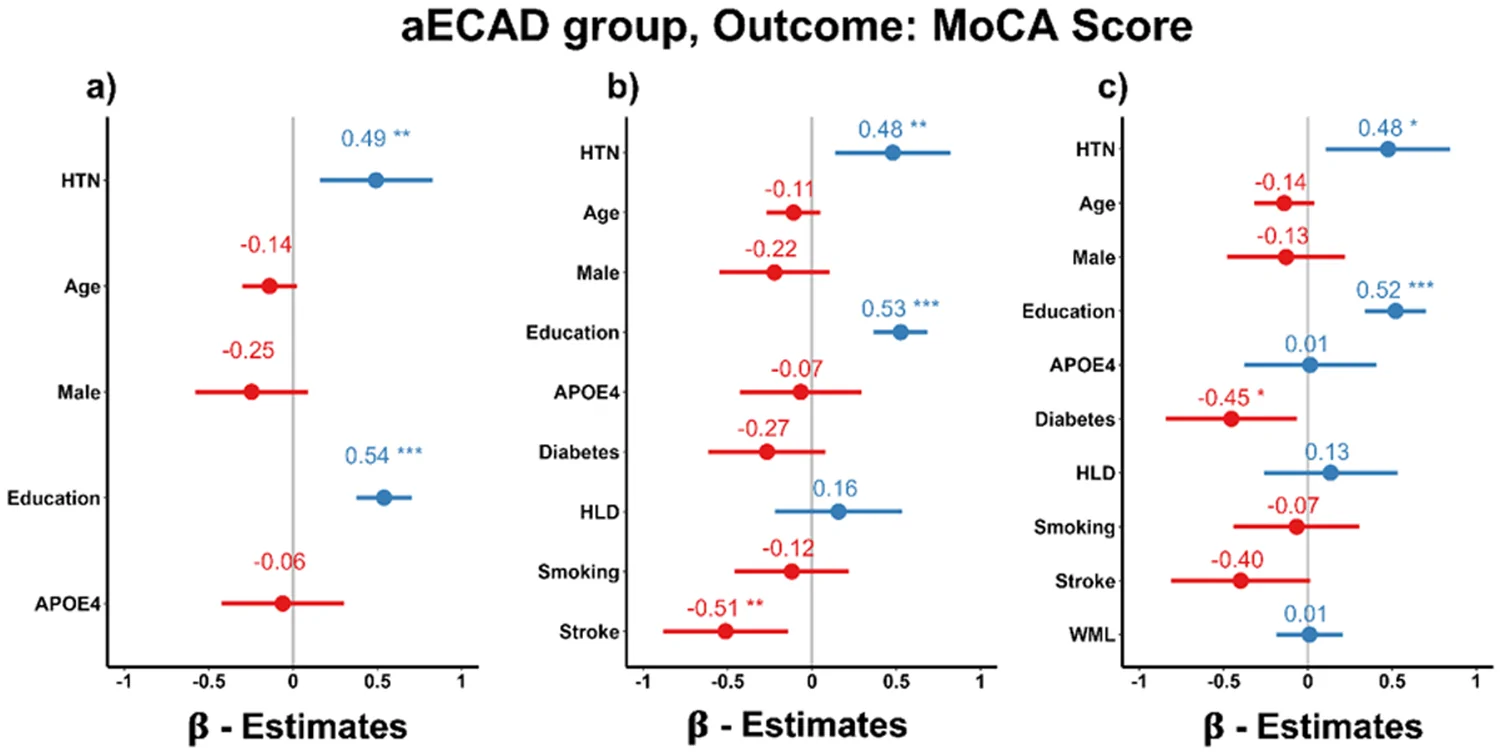
Parts a-c. In aECAD patients, HTN is associated with higher MoCA scores, CAM cohort. Multivariable linear regression models estimating the effect of HTN on cognition adjusting for **a)** age, sex, education and *APOE ε4 allele* **b)** demographics and vascular comorbidities **c)** and additional white matter lesion volumes. Abbreviations: *APOE ε4, Apolipoprotein ε4;* HTN, history of HTN; aECAD, asymptomatic extracranial carotid atherosclerotic disease; **β**, standardized beta estimates. *P<.05 **P<.01 ***P<.001

**Figure 3:**
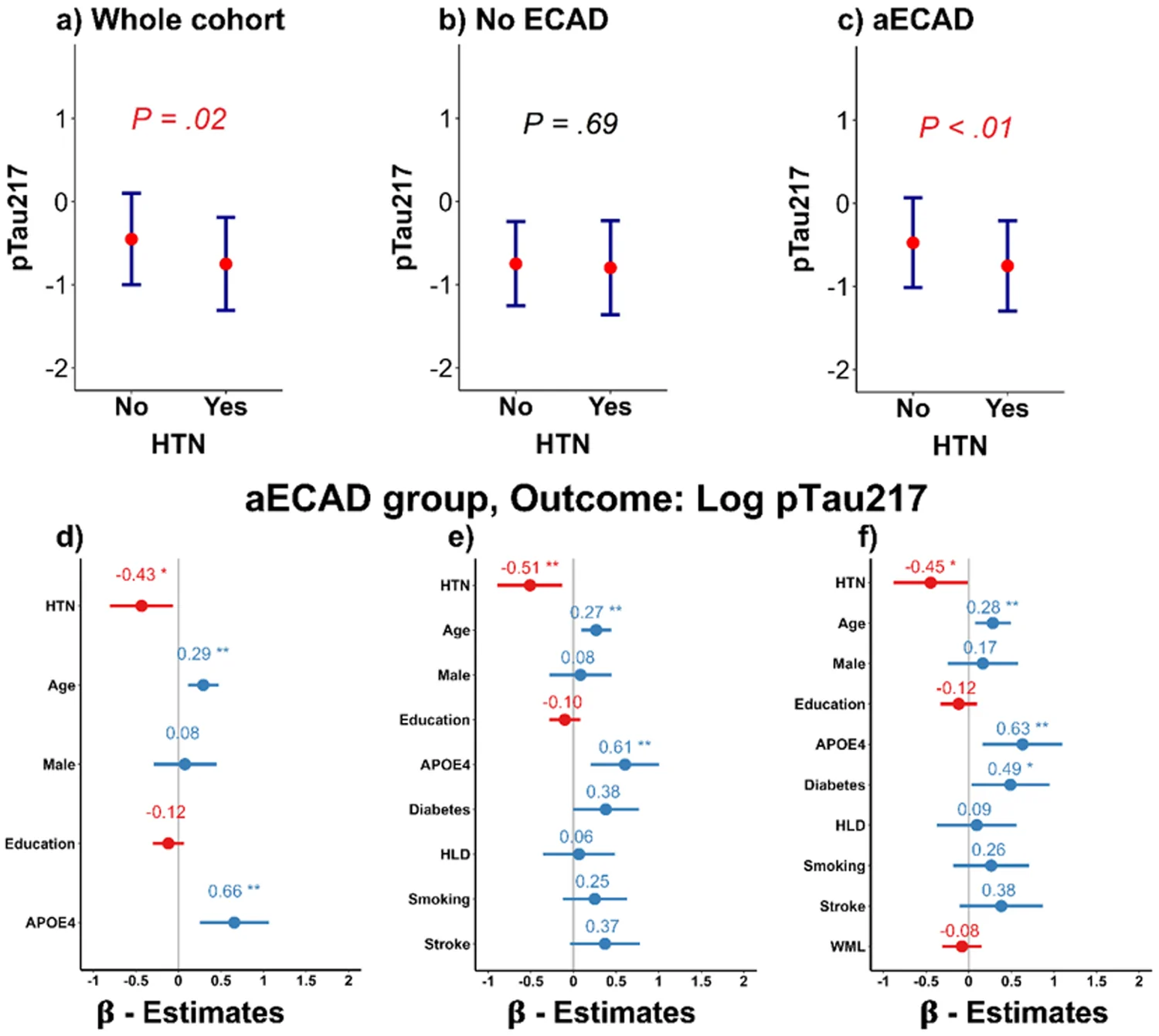
Parts a-f. In aECAD patients, HTN is associated with significantly decreased plasma pTau217 levels, CAM cohort. **a-c)** Mean plasma pTau217 scores were compared between those with HTN and without based on aECAD status. Mean differences were evaluated using Wilcoxon test. Plasma pTau217 levels were log transformed due to skewness. **d-f)**Multivariable linear regression models estimating the effect of HTN on pTau217 were analyzed. Abbreviations: *APOEε4, Apolipoprotein ε4;* HTN, history of HTN; aECAD, asymptomatic extracranial carotid atherosclerotic disease; log pTau217, log transformed phosphorylated Tau 217; **β**, standardized beta estimates. *P<.05 **P<.01 ***P<.001

**Figure 4:**
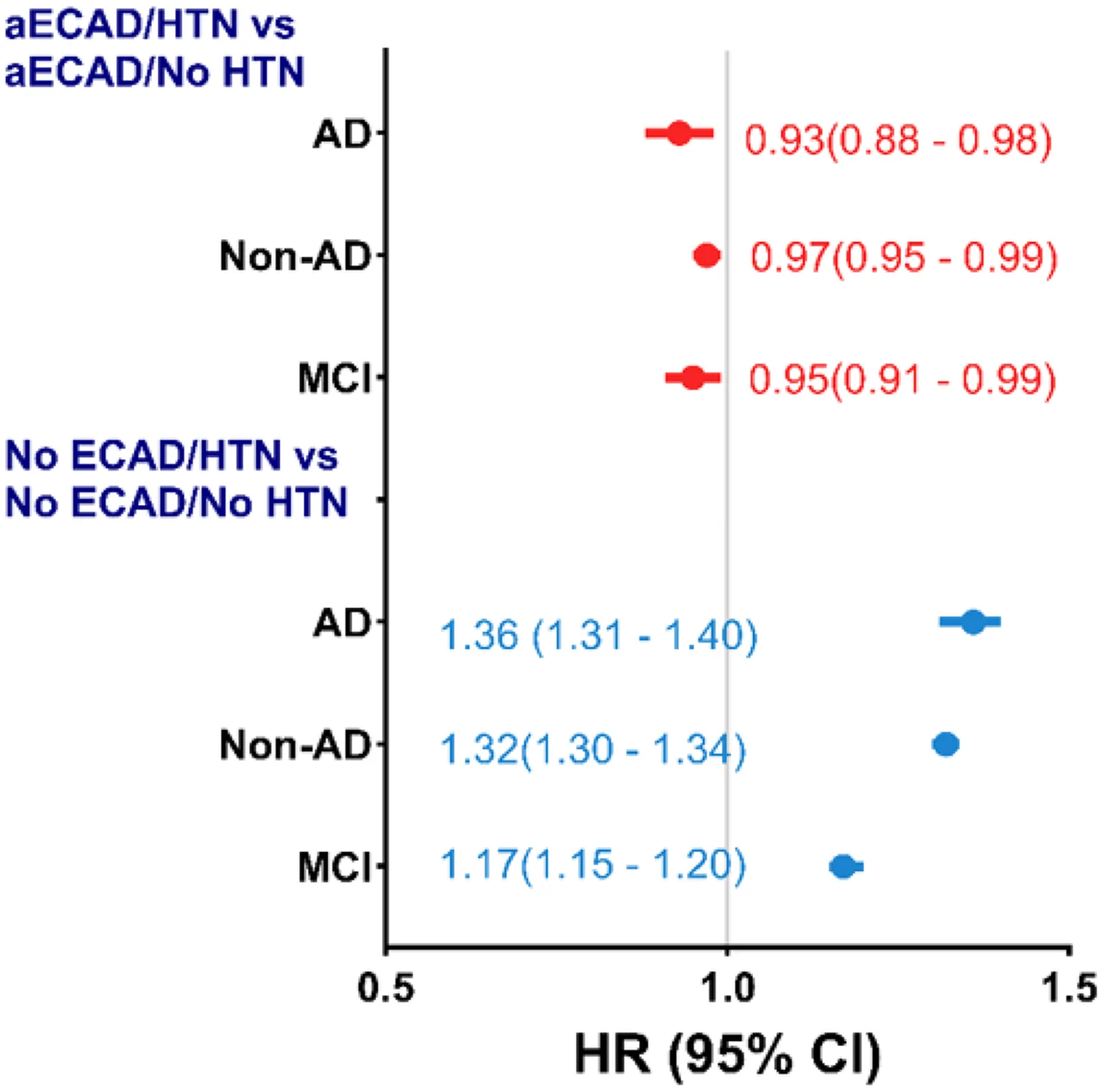
Comparative risk of cognitive outcomes by aECAD and HTN status, TriNetX cohort. Patients categorized into four exposure groups based on asymptomatic extracranial carotid artery disease (aECAD) and HTN (HTN) status were followed up to 20 years after index diagnosis. Hazard ratios (HR) with 95% confidence intervals (CI) were calculated using Cox proportional hazards models with censoring applied. For each outcome, the groups were propensity-score matched for relevant covariates, and the outcome was compared between the matched groups. Each eligible individual was followed up from the index event until the occurrence of the outcomes, death, loss to follow-up, or 20 years after the index event, whichever occurred first. HTN was associated with reduced ADRD risk in patients with aECAD but increased risk in those without aECAD; the corollary was also true that aECAD was associated with lower ADRD risk in hypertensive patients but higher risk in non-hypertensive patients. AD, Alzheimer's dementia; Non-AD, Non-Alzheimer's dementia; MCI, Mild Cognitive Impairment.

**Figure 5:**
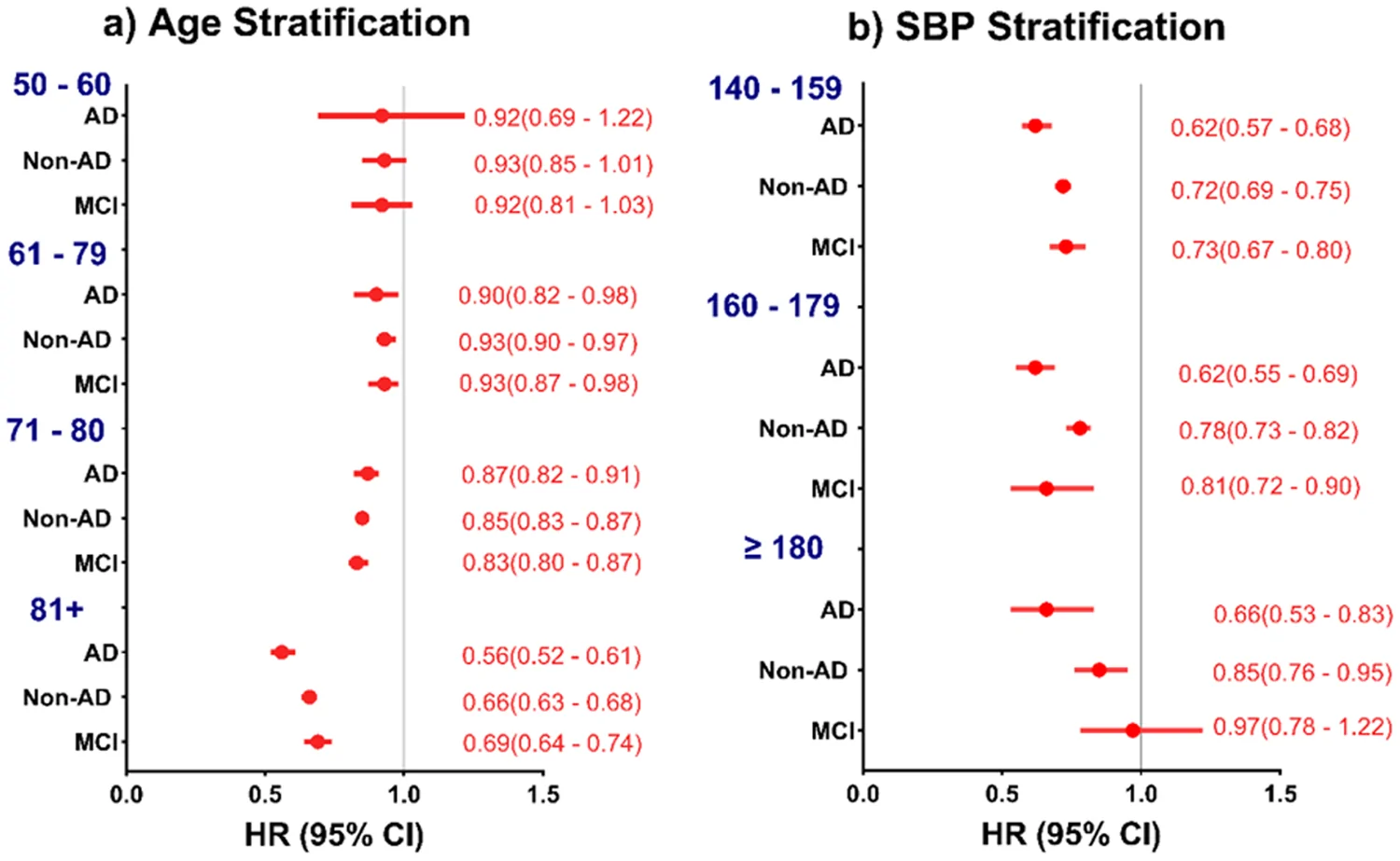
Parts a-b. Comparative risk of cognitive outcomes in aECAD patients with and without HTN stratified by age and SBP, TriNetX cohort. a) For the age-stratified analysis, patients with aECAD and HTN were compared with those with aECAD without HTN within age bands of 50-60, 61-70, 71-80, and 81+ years at index aECAD diagnosis. Risk of ADRD decrease significantly with increasing age within aECAD/HTN group. b) For SBP stratified analysis, patients who were 81+ years with aECAD/HTN and various SBP ranges were compared to aECAD/No HTN patients. These data demonstrate that protective effects of HTN diminish with more severe elevation of blood pressure in 81+ cohort. HR with 95% CI were calculated using Cox proportional hazards models with censoring applied. For each outcome, the groups were propensity-score matched for covariates related to the outcome, and the outcome was compared between the matched groups. Each eligible individual was followed up from the index event until the occurrence of the outcomes, death, loss to follow-up, or 20 years after the index event, whichever occurred first. aECAD, Asymptomatic extracranial carotid atherosclerotic disease; AD, Alzheimer's dementia; CI, Confidence interval; HR, Hazard ratio; MCI, Mild Cognitive Impairment; Non-AD, Non-Alzheimer's dementia; SBP, Systolic blood pressure.

**Table 1:** Carotid endarterectomy mitigates the protective effects of HTN in aECAD Patients, TriNetX Cohort.

Cohort	Outcomes	aECAD/ CEA/HTN, n (%)	aECAD/ CEA/No HTN, n (%)	HR (95% CI)
Age70-80	AD	143 (2.1%)	145 (2.2%)	0.87 (0.69–1.10)
(n = 6,748)	Non-AD	685 (10.2%)	664 (9.8%)	0.92 (0.83–1.02)
	MCI	230 (3.4%)	195 (2.9%)	1.05 (0.87–1.27)
Age 81+	AD	38 (2.9%)	37 (2.8%)	0.85 (0.54–1.34)
(n = 1,317)	Non-AD	161 (12.2%)	180 (13.7%)	0.74 (0.59–0.91)
	MCI	44 (3.3%)	38 (2.9%)	0.95 (0.62–1.47)

These data evaluate the effect of HTN in patients with aECAD and CEA. Each group was propensity score matched and had a mean follow-up of 3.0 ± 2.1 years in the 81 + cohort and 4.8 ± 3.3 in the 70–80 cohort. These data demonstrate that in patients with aECAD/HTN with CEA there is no longer a significant difference in dementia outcomes compared to those with aECAD without HTN. This indicates that in aECAD patients with CEA, there is no longer a protective effect of HTN.

BBB, blood brain barrier; CI, confidence interval; HR, hazard ratio; aECAD, asymptomatic extracranial carotid artery disease; HTN, HTN; CEA, carotid endarterectomy; AD, Alzheimer’s dementia; MCI, mild cognitive impairment, non-AD, non-AD dementia.

## Data Availability

The deidentified data utilized in the analyses for this study will be made available to qualified researchers upon reasonable request to the corresponding author (CW) and receipt of a signed data access agreement form.
